# What Surgeon Should Know about Probiotics

**DOI:** 10.3390/nu14204374

**Published:** 2022-10-19

**Authors:** Katerina Kotzampassi

**Affiliations:** Department of Surgery, Aristotle University of Thessaloniki, 54636 Thessaloniki, Greece; kakothe@yahoo.com

## 1. Introduction

Back in the 1980s, Fuller R, when working on gut flora, concluded that “there is good evidence that the complex microbial flora present in the gastrointestinal tract … is effective in providing resistance to disease; however, the composition of this protective flora can be altered by dietary and environmental influences, making the host susceptible to disease” [1]. He coined the term *probiotics,* “live microbial feed supplements, which beneficially affects the host by improving its microbial balance”, which “can be seen as a means of repairing deficiencies in the flora induced by dietary and environmental stress”, and he insightfully continues: “when the way probiotics work is known, we will be able to take a more rational approach to the selection of strains used” [2].

Today, 40 years later, it is perhaps time to discuss how far we really know about the “secrets” of probiotics and what it is we really need to know in order to use them efficiently and effectively.

## 2. Why Meta-Analyses Have Failed to Provide Strong Evidence

Even for common medical conditions, such as antibiotic diarrhea, with tens of thousands of cases, it is difficult for meta-analyses to show a strong positive effect for probiotics—even more so following an operation or severe trauma. Dissimilar populations, different microbiomes, different surgeries and different probiotics make comparisons difficult.

The physical trauma of surgery, augmented by the stress of anesthesia, pharmacologic manipulation (opioids, antibiotics, and acid-suppressing agents), alterations in tissue perfusion and pH, perioperative nutritional strategies, and the deprivation of gut motility, have been shown to cause gut microbial dysbiosis, inflammation and harmful disruption of the epithelial barrier, allowing so-called “bacterial translocation” in parallel with the depression of the host immune defense system [3,4,5]. Within this wide-ranging context, both the general health status of the patient scheduled for surgery and their own microbiome are of particular interest [6], while the most important pitfall remains the extensive heterogeneity in patient populations, the disease processes, different surgeries which may differently influence the gut microbiome (involving the colon or otherwise, clear operations such as hernia repair versus acute viscera inflammations, the presence of cirrhotic or congestive liver or obstructive jaundice), and treatment modalities in surgical settings [7,8].

Additionally, apart from all the above, most of the systematic reviews have reported substantial heterogeneity among the different probiotic regimens used, varying in: species and strains, the number of species and strains used, the amount of bacteria (cfu) administered, the administration route, the duration of treatment and the protocol applied for administration [7,9,10], all of which limit the ability to draw robust clinical conclusions to establish an optimal prophylactic treatment or at least to make recommendations on the best regimens, strains, dosages, and length of treatment [7,10,11,12].

In the most recently published systematic review (36 RCTs with 3305 oncological patients plus six non-randomized/observational cohort studies), the authors finally “failed” to pool results in a meta-analysis since—besides the substantial heterogeneity among interventions—21 diverse probiotic formulas were evaluated. Thus, their findings once again support the current thinking that probiotic effects are specific to the product/formulation [13].

## 3. Single Strain or Multi-Strain?

The dilemma “single strain or multi-strain” has already been more or less resolved: multiple stains offer increased benefits, since it is well-known that each specific strain works in a different way [14]. Recent evidence of this comes from an experimental study on trauma, where the well-recognized healing properties of *L. plantarum* UBLP-40 were finally found to be inferior for tissue repair compared to a mixture of *L. rhamnosus* UBLR-58 plus *B. longum* UBBL-64 [15]. However, there are many papers with encouraging positive results dealing with one strain only, such as those by Bengmark using *Lactobacillus plantarum* 299 plus oatmeal for over a decade in the 1990s [14].

The future seems to be towards multi-species formulas, designed after careful consideration of the properties and way of action of each individual probiotic strain and how they best meet the specific requirements of the particular patient group. In other words, a different probiotics regimen should probably be given depending on whether the priority is, for example, wound healing or the avoidance of postoperative pneumonia.

## 4. Preoperatively, Postoperatively, Perioperatively or All Together?

It is desirable for the patient to enter the stressful surgical procedure with a healthy gut microbiome—not forgetting the body’s other microbiomes, which may also not be healthy: that of the nose, from, say, a common cold; the oral cavity (dental decay, periodontitis), the upper respiratory tract, and the skin (acne, psoriasis and similar). All these dictate the preoperative probiotic treatment for microbiome restoration. However, this could perhaps only be possible in cases of elective surgery, and not in emergency operations or severe trauma, cases where it would be practically more useful.

Additionally, how many days should treatments be administered for? According to an old belief, probiotics should be given for a minimum of 14 days, giving them the time to colonize the mucous membranes [14]. In a recent meta-analysis of 15 RCTs on colorectal surgery, the authors [16] recommend they be given for 7 days pre- and 7 days postoperatively; however, by analyzing each RTC, the duration of treatment showed large fluctuations, from 15d pre- to 30d postoperatively, with the total duration ranging from 30 to 7 days—the median being 16 days. An informal personal survey in the results of one study of those included in this meta-analysis, having the longest duration of administration, 33 days, and at the same time one of the most abundant probiotic combinations [n = 8], revealed that it also had the highest rate of surgical site infections (28.2% versus 35.9% in the control group; p, 0.682) [16].

On the other hand, we must not overlook the fact that, for the colon cancer patients having received oral probiotics preoperatively, these are “washed-away” when undergoing bowel preparation for colonoscopy and/or colon surgery. Multiple studies have reported vast disturbances in microbial counts and diversity following these procedures, which may themselves create microbiota disturbances with health consequences [17,18].

## 5. Topical or by Mouth, or Both?

Each individual hosts a unique skin microbiome that fluctuates daily, based on its contact with surfaces, diet and medications received, as well as the possible stress and surgical procedures they are subjected to [19,20]. This microbiome constitutes the skin barrier from external threats, functioning to promote local homeostasis by means of upregulating the secretion of several defensive biomarkers, which in turn boosts the skin’s immune function [19,21]. After surgically induced trauma—as is true for other causes, the only difference here being that it is clear cut and theoretically sterile—skin continuity is disrupted; this is followed by the wound healing process to prevent the entrance of harmful microorganisms, and restore tissue continuity and thus re-establish skin barrier function [15].

Topically applied probiotics may have the potential to inhibit the formation of biofilm over wounds [21]. In vitro, the addition of probiotics to pathogenic bacterial cultures can inhibit the formation of biofilm development by pathogenic bacteria and fungi by about 50% [22], while a local injection of *L. plantarum* in full-thickness burn wounds of rabbits challenged with *P. aeruginosa* resulted in decreased severity and length of *Pseudomonas* infection, compared to the control vehicle [23]. In parallel, they interact with the host and/or bacterial cells and inhibit infection-secreting signaling factors [24] by a species-specific antagonism and through the regulation of antimicrobial peptides.

The topical application of probiotics on skin wounds of any kind seem to positively stimulate the wound healing process, as evidenced from in vivo and in vitro, as well as from clinical studies [19,25,26]. Wounds treated with *Lactobacilli* showed a continuous augmentation of neutrophil and macrophage numbers, by means of increased cytokine and chemokine release [27,28], which results in the intensification of the inflammation process, while finally shortening the early/inflammatory phase of the wound healing process, as has been documented in vivo with *L. plantarum* [15,29]. Furthermore, probiotics, especially from the *Lactobacillus* spp. genus, as is *Lactobacillus casei Shirota*, *L. brevis* and *L. plantarum*, can stimulate the migration and proliferation of fibroblasts—which restore the wound area by providing collagen in the new extra-cellular matrix—and the formation of new blood vessels through the involvement of endothelial and perivascular cells [30,31]. Additionally, some bacteriocins lead to significant neo-vascularization and cell migration, aiding in forming a thick epithelial layer [32].

In an experimental study on rats subjected to oral mucosa trauma, the topical administration of probiotics for seven consecutive days produced the highest number of fibroblasts and blood vessels compared with the rats receiving them orally [31]. Fibroblasts and blood vessels play an important role in the proliferation phase, primarily to correct defects in the wound area by providing collagen to the new extracellular matrix [19,33].

A possible underlying mechanism of topical action seems to be through ketatinocytes, which can activate a pathway for probiotics to provide a beneficial mechanism of action for the host, that is, through the activation of Toll-like receptors (TLRs), inducing the production of chemokines and cytokines, such as IL-8, which in turn will stimulate the re-epithelialization process, angiogenesis, and the formation of the extracellular matrix [26,34,35].

On the other hand, the systematic administration of probiotics can improve the wound healing process by increasing the regulation of oxytocin in the gut–brain–skin axis, through the vagus nerve pathway [36], leading to the rapid deposition of collagen, which is essential for proper wound healing [37].

Since the mechanisms of action between oral and topical probiotics are different, oral administration, which also protects against other complications, can safely be combined with topical administration, which acts both protectively against possible wound infection and accelerates wound healing. Moreover, the ideal would be for the topically administered probiotic to be a combination of two or more strains, which have some further “specialization” in different stages of the healing process [15].

Of course, there are no such studies yet. However, the use of probiotics has no contraindications, nor does it have any side effects. After all, more than a few of us have sprinkled a probiotic capsule on an infected surgical wound, just as we once did with antibiotics. There is no official indication and no guidelines, but there is also no contraindication. Time and use will finally prove if there is any benefit.

## 6. Conclusions

Instead of another conclusion, I will quote the proposals of the International Scientific Association for Probiotics and Prebiotics (ISAPP) organized consensus meeting of clinical and scientific experts on the mechanisms underlying probiotic effects [38]. They suggest three possible mechanisms of action: the “widespread”, the “frequent”, and the “rare”. The widespread designation is for those common among probiotics—colonization resistance; acid and SCFA production; the regulation of intestinal transit; the normalization of perturbed microbiota; the increased turnover of enterocytes; and the competitive exclusion of pathogens. In the frequent category are the mechanisms observed among different species—vitamin synthesis; direct antagonism; gut barrier reinforcement; bile salt metabolism; enzymatic activity; and the neutralization of carcinogens. Finally, the rare ones are strain-specific mechanisms—neurological, immunological, and endocrinological effects and the production of specific bioactives.
